# Importance of parental anxiety in management of developmental breast conditions in children: A study with a prospective hierarchical regression model

**DOI:** 10.1097/MD.0000000000038514

**Published:** 2024-06-14

**Authors:** Mustafa Yaşar Özdamar, Şenol Biçer

**Affiliations:** a Department of Pediatric Surgery, Faculty of Medicine, Erzincan Binali Yildirim University, Erzincan, Turkey.

**Keywords:** anxiety, breast, children, management, parent

## Abstract

The parent is the most critical link and decision-maker between the patient and the healthcare provider in treating many pediatric diseases. This entity is essential for the management of pediatric breast diseases for which the rate of surgical intervention is known to be very low. Although previous publications have emphasized that pediatric breast diseases may cause alarming anxiety in parents, the demographic factors that influence this anxiety have not been investigated. Even if practitioners complete patient management with appropriate procedures, treatment is incomplete if the questions remain unanswered. In this observational prospective study, we investigated the demographic factors that affect parental anxiety, which should be prioritized to prevent incomplete management. The Beck Anxiety Inventory score (BAS) created by the parents of 409 boys and girls aged 0 to 17 with breast conditions was recorded at the diagnosis, termination of treatment, and final control stages. A 2-stage hierarchical logistic regression model was applied to show how strongly the demographic characteristics of parents and their children predicted the parental BAS. Of the demographic characteristics, there was a significant correlation (*P *< .05) between the patient’s sex, age, developmental period, Tanner stage, referral status, management method, family’s place of residence, economic distress, and BAS. However, according to the 2-stage hierarchical regression model, only 3 demographic characteristics, the patient’s gender, place of residence, and method used in patient management, significantly predicted BAS (*P *< .05, Δ*R*^2^ = .35). Among the many factors that affect anxiety experienced by parents whose son or daughter has breast problems, the gender of the child, place of residence of the family, and management methods used by the practitioner are demographic characteristics that should be taken into consideration.

## 1. Introduction

According to the literature to date, breast diseases in children of both sexes have been successfully managed by practitioners. However, breasts, which stand out as a sex characteristic in the female population, especially during adolescence, have been the subject of more studies than have boys. In a patient presenting with breast complaints, after carefully taking the patient’s history in the first stage, the physical examination begins in the second stage. In the second stage, whether there are features other than normal breast tissue appearance and palpation findings is determined. The third stage involves radiological imaging. If a mass is detected in the breast after the examination or if there is a suspicious lesion in adjacent structures, such as an armpit or a superficial mass in the chest, the first examination to be used is ultrasonography. Confirmation of suspicious lesions using pre-interventional biopsy or direct surgical intervention is the next stage in childhood breast disease. In other words, in the fourth stage, diagnosis by ultrasonography supports the examination and the surgical branch makes the final decision for surgery. As shown in previous studies, there is a strong possibility that the excision material is benign in most cases. In contrast to the meager rates of malignant breast pathological results (below 0.1 per hundred thousand), the rate of benign breast masses, which is 3%, has not been considered to be very high by breast specialists.^[1–3]^

The youngest age group included newborn patients. This study revealed that parental anxiety is widespread in couples having a baby for the first time. For example, in the neonatal period of breast hypertrophy, parents get into evil thoughts by associating the condition of their baby, who was born a few days ago, with the breast disease in adults. Explaining parents that their baby’s breast problem is a temporary condition caused by estrogen passing through the placenta during the intrauterine period and that periodic follow-ups are sufficient will reassure them.^[1–4]^ However, even if not deemed necessary, breast imaging using ultrasonography increases the assurance that there are no foul situations. The presence of lymph nodes in the armpit area of an underweight child, especially after viral infections, worries the parents. As most parents are aware of the relationship between breast cancer and the armpit lymph nodes in adults, they are worried about the same situation in their children. However, when the specialist consulted by the parent detects 3 to 4 mm lymph nodes that may be postinfection on ultrasonography and explains that this situation is temporary, the intensity of anxiety decreases. Similarly, parental anxiety has also been mentioned in other common pediatric physiological and pathological breast complaints such as asymmetrical normal breasts, breasts with osseous structures (pectus excavatum and carinatum, Poland syndrome), ectopic breast tissue, gynecomastia, cysts, hematomas, and benign masses.^[1–3,5,6]^

Although previous publications have emphasized that pediatric breast diseases may cause alarming anxiety in families, parents, and patients,^[2,7]^ influential demographic factors have not been investigated. In this observational prospective cohort study, we investigated the demographic factors that play essential roles in parental anxiety across 5 Tanner stages of physiological and pathological pediatric developmental breast conditions from initial presentation to termination of treatment. We aimed to present demographic factors that practitioners can prioritize to maintain parental anxiety at minimum levels.

## 2. Materials and methods

This prospective cohort study, including pediatric patients presenting with physiological or pathological breast complaints at our pediatric surgery outpatient clinics from April 2015 to April 2023, was performed after approval by the Ethics Review Committee of the University (Approval: 2015-48; 24/01). The privacy and ethical research objectives of this study were in accordance with national laws and the Declaration of Helsinki. The study included 409 participants aged 0 to 17 years and their parents. Participants or their parents in the study group were excluded if they had malignant, psychiatric, or chronic disease. Practitioners identified eligible patients. The patients’ parents received assistance from a practitioner during data collection, principally to help them complete the questionnaire. Written informed consent was obtained from the parents of all children.

### 2.1. Demographics of pediatric subjects with breast complaints

#### 2.1.1. Developmental characteristics of pediatric subjects

The patients were divided into 2 cohorts based on their stage of life: prepubertal period (including newborn, infancy, toddlerhood, early childhood, and middle childhood; “0–11 years”); and pubertal period (12–17 years). Body mass index (BMI) was calculated by dividing weight (in kilograms) by height (in meters squared) according to the patient height and weight by the outpatient staff.^[8]^ Patients were considered healthy, underweight, or obese according to their weight and BMI as follows: underweight, less than the 5th percentile; average weight, between the 5th and 84th percentiles; and overweight, greater than or equal to the 85th percentile. The Tanner stages (TS) of normal breast development were based on the clinical appearance of the developing breast and the Tanner stages of the normal breast. As the ultrasound appearance of the breast at various Tanner stages has been well described, ultrasound has been used for breast evaluation in radiological imaging.^[9]^

#### 2.1.2. Follow-up and treatment methods of pediatric subjects

Four different methods were used in the follow-up of patients from their admission to our center until their treatment was concluded. The methods were “only follow-up of the patient” (Method 1), “follow-up with ultrasound” (Method 2), “follow-up with ultrasound adding medication” (Method 3), and “surgery-added follow-ups (Method 4).

#### 2.1.3. Physiological and pathological breast complaints of pediatric subjects

The breast complaints of the patients were grouped under suitable headings: lymph node, asymmetric normal breast, breast with osseous structure (e.g., pectus excavatum and carinatum), ectopic breast tissue, gynecomastia, cyst (or fibrocystic changes), hematoma, abscess or mastitis, galactocele, galactorrhea or Witch milk, and benign mass (fibroadenoma).

#### 2.1.4. Referral status on admission of pediatric subjects

As a factor affecting the patient, the parent, and our treatment method, whether the patient was referred to our hospital from another center was questioned and recorded in addition to demographic data.

### 2.2. Demographics of parents

Although all pediatric patients in the study presented with one of their parents, most applied with their mothers (95%). However, a form in which the parents answered together was used in the survey. Demographic information regarding place of residence, economic status, and educational level, which affect the socioeconomic life of parents, was collected and recorded.

This study used the Beck Anxiety Inventory (BAI), which is a questionnaire used to measure the severity of anxiety symptoms in adults. This survey consisted of 21 items. Each item is scored between “0” (no symptom) and “3” (symptoms always present). The Beck Anxiety Inventory total score (BAS) was calculated from the total score of all items as minimal (0–7), mild (8–15), moderate (16–25), and severe (26–63) anxiety scores. To parents of pediatric individuals monitored and treated with 1 of the 4 management methods mentioned in section 2.1.2, the BAI test was administered 3 times: at admission (BAS 1), at treatment termination (BAS 2), and at final follow-up testing (BAS 3).

### 2.3. Statistical analysis

Categorical data were reported as numbers and percentages, whereas continuous data were reported as mean ± SD. A multivariable logistic regression model was used to evaluate the association between parental anxiety and the demographic characteristics of both pediatric subjects and parents. Additionally, the demographic data of the pediatric participants and their parents were used in a 2-stage hierarchical regression analysis to determine which variables significantly and strongly predicted the BAS-dependent variable. In the first stage, data on pediatric subjects, including sex, age, developmental period, Tanner stage, BMI category, referral status, breast complaints, and patient follow-up method, were entered into the analysis. In the second stage, hierarchical regression analysis was performed by adding parental data, including place of residence, educational level, and economic status, to the first-stage data.

Finally, the parents’ BAS scores of 1, 2, and 3 were compared using a paired-sample *t* test to evaluate whether there was a significant change in the scores in these 3 stages. Whether patient sex showed any differences in BAS changes was also assessed using an independent-sample *t* test. Statistical analyses were performed using SPSS (version 16.0; SPSS Inc., Chicago, IL, USA).

## 3. Results

### 3.1. Results of demographic characteristics of pediatric subjects

Descriptive characteristics of the participants and their parents are presented in Table 1. The 409 children aged between 1 month and 17 years included in the analysis were, on average, 12.38 ± 4.58 years old, and 71.6% (n = 293) of the patients were girls. Of the patients, 323 (71%) were in the pubertal developmental period, 86 (21%) were in the prepubertal developmental period, and all patients in the prepubertal period were in the TS 1 breast development stage. Regarding BMI, 173 (42.3%) patients were average weight, 160 (39.1%) were overweight, and 76 (18.6%) were underweight. Among patients who visited our outpatient clinic for pediatric surgery, 347 (84.8%) were referred from family medicine and pediatric outpatient clinics.

**Table 1:** Demographics of pediatric patients and parents.

Variables		N	%
Sex	Boy	116	28.4
	Girl	293	71.6
Developmental period	Prepubertal	86	21.0
	Pubertal	323	79.0
Tanner stage (TS)	TS1	86	21.0
	TS2	18	4.4
	TS3	80	19.6
	TS4	137	33.5
	TS5	88	21.5
Referred patient	No	62	15.2
	Yes	347	84.8
Complaints	Lymph node	36	8.8
	Asymmetric normal breast	157	38.4
	Breast with osseous structure	50	12.2
	Ectopic breast tissue	13	3.2
	Gynecomastia	66	16.1
	Cyst and fibrocystic changes	24	5.9
	Hematoma	11	2.7
	Abscess or mastitis	24	5.9
	Galactocele, galactore or Witch milk	18	4.4
	Benign masses (fibroadenoma)	10	2.4
BMI category	Normal (Average)	173	42.3
	Underweight	76	18.6
	Overweight	160	39.1
Place of residence^a^	Urban	309	75.6
	Rural	100	24.4
Educational level^a^	Primary school	92	22.5
	High school	141	34.5
	University	176	43.0
Economic distress^a^	No	124	30.3
	Yes	285	69.7
Method	Follow-up	145	35.3
	Follow-up with USG	225	55.1
	Follow-up with medication (+), (−)USG	21	5.1
	Surgery-related follow-up	18	4.4
Total	For each variable	409	100.0

a Demographics of parents. (The others belong to patients admitted with breast complaints.)

Considering the 2 genders, breast complaints, in order of frequency, were asymmetric normal breast (38.4%), gynecomastia (16.1%), breast with osseous structure (12.2%), benign masses (fibroadenoma, 2.4%), lymph nodes (8.8%), cysts (5.9%), abscess or mastitis (5.9%), galactocele, galactorore or Witch milk (4.4%), ectopic breast tissue (3.2%), and hematoma (2.7%). Among children’s breasts with osseous structures, the majority of asymmetrical breasts associated with pectus excavatum and carinatum (85%) were associated with minimal rib lateralization in both boys (35%) and girls (75%).

#### 3.1.1. Results of follow-up and treatment methods of pediatric subjects

The follow-up procedure and duration of the cases are summarized in Table 2. The average follow-up period of the pediatric subjects was 71.1 (18–153) days. A 145 (35.3%) children were followed up with Method 1, and their treatment was terminated. The majority of these were patients with asymmetrical breasts. Of the 4 methods used from the first application of the patients to the follow-up and termination of treatment, “follow-up with ultrasound” (225 patients, 55%; Method 2) was used the most. Cysts (including fibrocystic changes), hematoma secondary to trauma, gynecomastia, asymmetry in the breast, and detection of axillary lymph nodes were the cases in which method 2 was most frequently used methods. In Method 3, which was applied with or without ultrasound when making the diagnosis, 24 (5.1%) patients were treated with medication for mastitis or breast abscess, especially during neonatal or adolescence.

**Table 2:** Follow-up and treatment methods of patients.

Compliant or disease	n	%	M1	M2	M3	M4	FD
Lymph node	36	8.8	36	a			35
Asymmetric normal breast	157	38.4	57	100			152
Breast with osseous structure	50	12.2		50			65
**Ectopic breast tissue**	**13**	**3.2**		**8**		**5**	**95**
Gynecomastia	66	16,1	45	21			45
**Cyst**	**24**	**5.9**		**14**	**8**	**2**	**153**
Hematoma	11	2.7		11			18
**Abscess or mastitis**	**24**	**5.9**	**6**		**11**	**7**	**34**
Galactocele, galactore or Witch milk	18	4.4	1	15	2		27
**Benign masses (fibroadenoma**)	**10**	**2.4**		**6**		**4**	**123**
Total	409	100	145	225	21	18	71.1*
%	100		36	55	5	4	

M: Follow-up treatment method. (M1: Those whose treatment was terminated with follow-up only. M2: Ultrasonography was added to M1. M3: Medical treatment added to M2. M4: Surgical intervention was added to M3.)

Possible candidate patients for surgical intervention are available in the **dark rows**.

FD: Follow-up duration (the period between follow-up, treatment, and last control day).

* Average follow-up time (day). a: Some patients underwent ultrasound at an external center and were excluded from this group.

Eighteen (4%) patients were followed up and treated using Method 4. As shown in Table 2, possible candidates for surgical intervention are available in dark rows (13 ectopic breast tissues, 24 cysts, 24 abscesses or mastitis, 18 galactoceles, and 10 fibroadenomas; 89 patients in total). Of the 89 patients, 71 aged > 18 years were referred to the general surgery department and 18 underwent surgery (Table 2).

#### 3.1.2. Verification of assumptions for regression analysis

**Figure 1.**
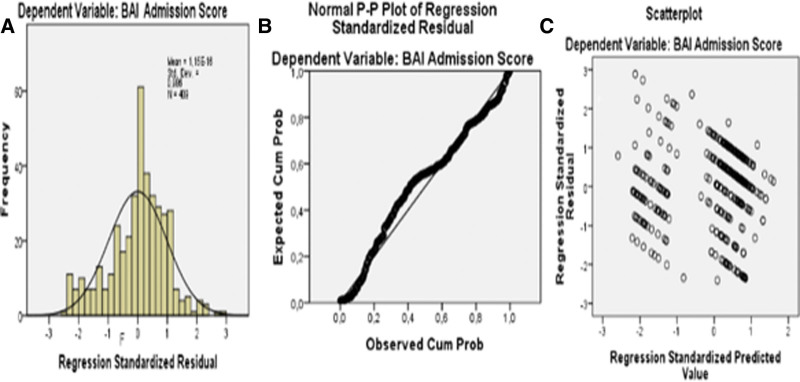
In the linear logistic regression analysis, scatter and residual plots showing predictive normality (A), linearity (B), and homoscedasticity (C) assumptions.

In the linear logistic regression analysis, scatter and residual plots showed that the predictive normality; linearity; and homoscedasticity assumptions were satisfied (Figure 1A–C). There was no multicollinearity between independent variables (*r *< .80). No extreme values were observed among the observed. Standard residual extreme minimum and maximum values were between (−) 2.40 and (+) 2.88. Cook distance value (.00) supports this assumption. The relationship between BAS and the mean of the dependent variables was linear. According to the regression analysis model, the Durbin–Watson coefficient was 1.66, and the residuals were independent. We calculated the odds ratio (OR) with a 95% confidence interval to analyze the relationship between the independent variables and the outcome of interest. The tests were 2-tailed with a significance level (*P*) of .05; *P < *.05 was considered statistically significant. According to the Collinearity Statistics, there was no multicollinearity problem (*VIF value < 8 and Condition Index (Cl) < 25*).

#### 3.1.3. The predictive power of independent variables in a multivariate regression model for parental BAS

The correlation (Pearson correlation, PC) and predictive significance between the demographic characteristics and BAS are presented in Table 3. The most substantial predictive variables for parental BAS were parental place of residence in the negative direction (*r=* −0.55; *P *< .05) and parental economic distress (*r =* +.44; *P* < .05) in the positive direction. A moderately strong correlation (.30 < *r *> .70) was observed. There was a weak (.09 *< r *< .25) and significant (*P <* .05) correlation between the pediatric subject variables sex, age, developmental period, Tanner stage, referral status and follow-up methods, and the BAS. No significant correlation (*P > *.05) was found between BAS and patient complaints, BMI, or parental education level.

**Table 3:** Correlation of demographic characters with each other and with BAS.

		BAS	Sex	Age	DP	TS	RP	C	BMI	PoR	EL	ED	M
PC	BAI admission score (BAS)	1.00	**0.25**	**0.12**	**0.12**	**0.10**	**0.09**	0.07	−0.02	**−0.55**	−0.02	**0.44**	**0.14**
	Sex		1.00	0.04	0.05	0.10	0.22	0.05	−0.20	−0.25	−0.01	0.22	0.15
	Age			1.00	0.91	0.88	0.04	0.01	0.11	−0.10	−0.23	0.14	−0.06
	Developmental period (DP)				1.00	0.84	0.07	0.06	0.09	−0.11	−0.29	0.09	−0.03
	Tanner stage (TS)					1.00	0.07	0.08	0.07	−0.09	−0.21	0.08	0.01
	Referred patient (RP)						1.00	−0.05	−0.14	−0.14	−0.05	0.20	0.07
	Complaints (C)							1.00	0.07	−0.03	0.00	0.01	0.25
	BMI category (BMI)								1.00	−0.02	0.12	−0.06	−0.15
	Place of residence(PoR)									1.00	0.05	−0.76	0.03
	Educational level (EL)										1.00	−0.02	−0.07
	Economic distress (ED)											1.00	0.01
	Method (M)												1.00
*P*	BAI admission score (BAS 1)	.	**.00**	**.01**	**.01**	**.03**	**.04**	.08	.36	**.00**	.37	**.00**	**.00**

PC = Pearson correlation.

Table 4 shows which variables significantly predicted parental BAS with the multivariate regression model. As the measurement units of the independent variables were different, β was used instead of the B coefficient; that is, the ability of the independent variables to predict BAS was evaluated according to standard error (SE). The multivariate linear regression model showed that the BAS-dependent variable was positively predicted (Δ*R*^2^ = 0.34; *F*-change =* *20.22; *P *= .00). Of all the independent variables with 34% predictive power, only 3 variables significantly predicted BAS (*P < *.05), including sex (*β =* .10), method (*β = *.16) in a positive direction, and place of residence (*β= −*.55) in a negative direction. However, when looking at the entire model, other parental and patient variables did not contribute significantly to the multivariate regression analysis (*P *> .05, Table 4).

**Table 4:** Multivariate linear regression predicting parent BAS.

Model Δ*R*^2^ = 0.34^a^				
	B	SE	β*	*P*
Sex	.43	**0.20**	**0.10**	**.03**
Age	.07	0.05	0.17	.18
Developmental period	.15	0.52	0.03	.77
Tanner stage	−.18	0.13	−0.13	.16
Referred patient	−.11	0.24	−0.02	.65
Complaints	.01	0.03	0.01	.77
BMI category	−.02	0.10	−0.01	.80
Place of residence	−2.60	**0.30**	−**0.55**	**.00**
Educational level	.11	0.11	0.04	.33
Economic distress	−.09	0.29	−0.02	0.75
Method	.44	**0.12**	**0.16**	**0.00**

a Dependent variable: BAI score admission (BAS).

* Since the measurement units of the independent variables were different, β was used instead of the B coefficient; that is, the ability of the independent variables to predict BAS was evaluated according to the standard error (SE).

#### 3.1.4. The predictive power of independent variables in a hierarchical regression model for parental BAS

Our study demonstrated relationships with more than one outcome by using a multivariate regression model. However, we also used a hierarchical regression model to predict the relationship more precisely than traditional predictions.^[10]^ For the hierarchical regression analysis, the demographic characteristics of children with breast complaints were entered into the first regression stage according to their importance in the literature (Model 1). The demographic characteristics of parents in the second model (Model 2) were included in the analysis. In the first stage of the regression analysis, the demographic characteristics of the patients explained BAS by approximately 9% and contributed significantly to the regression model (Δ*R*^2^ = 0.09, *F*-change = 5.20, *P *= .00). With the addition of parental demographic variables to Model 2, the predictive power of the variables for BAS increased significantly (Δ*R*^2^* = *.35, *F*-change = 54.63, *P =* .00).

In Model 1, the variables that positively and significantly contributed to predicting BAS were sex (*β = *.23; *P* = .00) and method (*β = *.12; *P* = .02). The other Model 1 variables did not significantly predict the BAS (*P* > .05). When parental variables were added to Model 2, although the contribution of sex and method variables decreased (β sex = .10, β method = .16), they continued to contribute significantly (*P <* .05) to the model. As in Model 1, the other patient variables did not predict substantially Model 2 (*P *> .05). Place of residence was one of the parental variables added to Model 2, and negatively and strongly predicted hierarchical regression analysis (*β=* −.55, *P* = .00). The other parental variables added to Model 2 did not contribute significantly to the model (*P > *.05).

#### 3.1.5. *Comparison of parental BAS after diagnosis, termination of treatment, and final follow-up examination (BAS 1, BAS 2, and BAS 3*) of *children*

The parental BASs of the patients were recorded at the first admission (BAS 1), termination of treatment (BAS 2), and control examination 1 month after the end of treatment (BAS 3). The variances were equally distributed according to Levene test (*P >* .05). A paired-sample *t* test revealed a significant difference between the BASs obtained in the 3 stages (Table 5; *P = *.00). Whether parental BAS varied according to patient sex was analyzed using an independent-sample *t* test. BAS 1 was significantly higher in girls’ parents (*P = *.00). There was no significant sex contribution to BAS scores of 2 or 3 (Table 6; *P > *.05).

**Table 5:** Comparison of parental BAS.

		Mean	SD	*P*
Pair 1	BAS 1 and BAS 2	5.49	2.36	**.00**
Pair 2	BAS 1 and BAS 3	8.96	2.18	**.00**
Pair 3	BAS 2 and BAS 3	3.47	1.79	**.00**

BAS: Parental BAI score (1: admission, 2: termination of treatment, 3: final test).

Pair: Paired-sample *t* test.

**Table 6:** Comparison of parental BASs according to patient gender.

	Sex^a^	n	Mean	SD	*P*
BAS 1	Boy	116	14.05	2.11	**.00**
	Girl	293	15.15	1.90	
BAS 2	Boy	116	9.26	1.55	.45
	Girl	293	9.39	1.53	
BAS 3	Boy	116	5.88	1.14	.98
	Girl	293	5.88	1.17	

BAS: Parental BAI Score (1: admission, 2: termination of treatment, 3: final test).

a Patient sex.

## 4. Discussion

Retrospective or prospective observational studies on childhood breast diseases have generally been conducted without including both the male child population and patient’s parents. Unlike previous studies, this prospective observational study included boys and parents of both genders. Although a consensus that would form a guideline could not be established in previous studies, patient management by practitioners supported each other. It has become an accepted entity that practitioners of childhood transient-developmental breast changes should collaborate with parents by improving their clinical knowledge and skills. In this context, reducing workups and costs, and preventing parental stress and anxiety have been shown to be one of the main goals. Although there are recommendations regarding parental anxiety in many studies that have yielded satisfactory results in patient approach, treatment, and follow-up in both transient-developmental and pathological breast conditions, especially in the female child population, no comprehensive investigation of parental anxiety has been conducted in patient management.^[2,7,11]^

As children age, their independence develops, interdependence between the child and parent increases, and the privacy of the child and their family begins to come to the fore. Social, cultural, economic, psychological, and educational parameters are essential for mutual interactions within a family.^[12]^ Parrish et al stated in their studies that the parent is the most critical link between the patient and practitioner for a successful outcome in treating pediatric patients. In line with these scientific anecdotes, previous publications have reported that although malignant breast disease in children is sporadic, the idea that their children may have permanent breast damage or suspicion of malignancy may cause psychological trauma and anxiety in parents who apply to relevant centers due to breast problems in their children.^[3,5,12,13]^ However, research is yet to be conducted on the demographic factors and consequences of parental anxiety.^[11]^ Therefore, we conducted this study to address this gap.

In our study, 293 of the 409 patients were female. We investigated the influence of the female sex on parental anxiety caused by breast conditions in children at admission (BAS 1), termination of treatment (BAS 2), and the last follow-up examination (BAS 3). While the effect of female sex showed a significant difference in BAS 1 (*P < *.05), it was not significant in BAS 2 and 3 (*P >* .05). However, for both sexes, using our patient follow-up method, the BAI scores decreased, creating a substantial difference (*P* < .05) between them (Tables 1, 5, and 6). This gradual and significant decrease in BASs demonstrates the suitability of the applied methods.

There was a significant weak correlation between parent BAS and most of the children’s demographic characteristics, including sex, age, referral status, method of follow-up, developmental period during which the number of pubertal children predominated in the study, and Tanner stage (Pearson correlations, .25 *< r *> .09 and *P < *.05). There was no significant correlation among BMI, children’s breast complaints, and BASs. In a study conducted by Nuzzi on adolescents with asymmetrical breasts, there was no correlation between BMI, current disease, and adolescent anxiety.^[14]^ Similarly, although parental anxiety was not evaluated in Nuzzi study, our study found no relationship between parental anxiety and existing breast complaints and the BMIs of parents’ daughters or sons. Among all the variables in this study, the features that were strongly correlated with BAS were the parental place of residence and economic status. Nuzzi et al^[14]^ also reported that differences in Tanner stages caused distress in adolescents. In our study, the correlation between the different Tanner stages of the parents’ children and parental anxiety was significant. A retrospective study suggested that inappropriate intervention and breast biopsy could be reduced by educating both the referring provider and patient’s family.^[5,11]^ In our study, no significant relationship was found between BAS and parents who were concerned about breast problems in their children, had high educational levels, or were aware of breast cancer (Table 3).

The dependent variable, BAS, was significantly correlated with most of the independent variables included in the study (Pearson correlation, *P *< .05). However, we examined how the working model predicts BAS using a holistic approach using regression models. In the multivariate regression model, of all independent variables with 34% predictive power, only 3 variables significantly predicted BAS: sex, method in a positive direction, and place of residence (urban area) in a negative direction (Δ*R*^2^ *=* .34, *P* < .05, Table 4). A 2-stage hierarchical regression analysis was performed to determine the association between demographic characteristics and BAS. In the first stage (Model 1), the demographic characteristics of the pediatric participants were included, and in the second stage (Model 2), parental characteristics were included in addition to those in Model 1. The results of this analysis were consistent with those of the multivariate regression analysis, with 3 pediatric subject variables and one parental variable significantly predicting BAS. However, while in Model 1, children’s demographic characteristics predicted BAS by 9%, in Model 2, with the addition of parent characteristics to child characteristics, the predictive power of the variables for BAS increased approximately fourfold to 35% (Δ*R*^2^* = *.35, *P* < .05, Table 7). The most substantial contribution to this result was “place of residence,” which predicted BAS with moderate power and a negative direction (*β *= −.55; *P *= .00). In other words, parents who were closer to the hospital and had more accessible checkups had lower BASs scores. It has previously been stated that patients referred to tertiary care centers come from rural areas or apply to relevant tertiary centers after being examined by more than 1 practitioner, creating anxiety for patients and their families.^[5,7,10]^ Consistent with these studies, anxiety in parents living in rural areas was more evident in the present study. Although patient referrals to our outpatient clinic from other centers showed a significant correlation with parental BAS, this did not contribute significantly to predicting BAS in our regression models.

**Table 7:** Hierarchical regression analysis predicting parents’ BAS.

		B	SE	β*	*P*
Model 1 Δ*R*^2^ = 0.09^a^	Sex	1.04	**0.22**	**0.23**	**.00**
	Age	.09	0.06	0.21	.14
	Developmental period	.28	0.59	0.06	.63
	Tanner stage	−.24	0.15	−0.17	.11
	BMI category	.07	0.11	0.03	.54
	Referred patient	.18	0.28	0.03	.51
	Complaints	.03	0.04	0.04	.48
	Method	.32	**0.14**	**0.12**	**.02**
Model 2 Δ*R*^2^ = 0.35^a^	Sex	.43	**0.20**	**0.10**	**.03**
	Age	.07	0.05	0.17	.18
	Developmental period	.15	0.52	0.03	.77
	Tanner stage	−.18	0.13	−0.13	.16
	BMI category	−.02	0.10	−0.01	.80
	Referred patient	−.11	0.24	−0.02	.65
	Complaints	.01	0.03	0.01	.77
	Method	.44	**0.12**	**0.16**	**.00**
	Place of residence	−2.60	**0.30**	−**0.55**	**.00**
	Educational level	.11	0.11	0.04	.33
	Economic distress	−.09	0.29	−0.02	.75

a Dependent variable: BAI score admission (BAS).

* Since the measurement units of the independent variables were different, β was used instead of the B coefficient; that is, the ability of the independent variables to predict BAS was evaluated according to the standard error (SE).

Previous studies have emphasized that the follow-up period for breast conditions can be 5 to 6 months, particularly for adolescents. In addition, it has been emphasized that in developmental breast conditions, it should not be left with the impression that their children need surgical intervention with medical statements that stress the family. In these studies, it has been insistently stated that patient follow-up with ultrasound is safe and sufficient.^[1–3,5–7,15]^ According to literature recommendations, in our study, BASs (BAS 1, 2, and 3) decreased significantly in the Method 2 group, where ultrasound was used in addition to patient follow-up (M2; n: 225, 55%). Invasive surgery (M4, not performed via biopsy) was performed in 18 (4.4%) of the 409 patients. We completed the management of 145 (36%) patients by monitoring them (M1), and 21 (5%) patients by treating them with medication in addition to ultrasound follow-up (M3). The follow-up period of the patients until the last follow-up was 71 days (range, 18–153 days; Table 3).

In this prospective observational study, we examined parental anxiety, which is an indicator of the attitudes of patients’ parents. Using the system previously recommended in the literature, we conducted 4 follow-up studies to evaluate developmental breast conditions in all age groups during childhood, considering parental attitudes. In the BAS study, parents’ first concern about their children was that they would associate their children’s breast conditions with those of adults. In a previous study, the most critical solution to this problem was to help parents make correct decisions by discussing the differences between adult breast disease and children’s disease. This is the essence of this study.^[6]^ That is, childhood breast problems have been resolved, but the other side of the coin is that reducing workups and preventing parental stress or anxiety have remained inadequate.

There was a significantly weak correlation between the parent BAS and most of the children’s demographic characteristics, including sex, age, referral status, method of follow-up, developmental period, and Tanner stage (*r *< .30, *P *< .05). There was even a moderately significant correlation between parental economic distress and the BAS (*r = *.44, *P = *.00). However, these variables did not significantly predict BAS in our regression models. On the other hand, prioritizing these demographic characteristics in patient management will alleviate the concerns of parents and the family’s most influential decision-makers. Interestingly, there was no significant correlation between BAS and parental education level, the nature of the child’s complaints, or BMI, and these variables did not significantly predict BAS in the regression analysis. In other words, the educational level of the parents who applied to our center due to their child’s developmental breast condition, their child’s weight, and the nature of the breast complaint did not significantly affect the BAS.

Although we evaluated the reflections of breast problems in children on parents with many parameters in this study, the results were explained in summary sentences. Such a topic may be the subject of more than 1 research under several headings. In other words, addressing each topic with summary explanations has been one of the limitations of our study. The most important limitation of our research was that family medicine, pediatrics, radiology, child development, and child and adult psychiatry specialists could not be included in this study. Broad-spectrum studies without these limitations are needed.

## 5. Conclusions

While most of the identified demographic characteristics were significantly correlated with parental anxiety, only 3 were significant predictors of parental anxiety in the regression analysis. Among these, parents’ residence in an urban area close to our center had the power to reduce BAS, and the patient, being a girl, had the power to increase BAS. Moreover, even if the most common and noninvasive method (follow-up with ultrasound) was used, the techniques used in patient management significantly predicted an increased BAS. Such observational prospective studies with larger cohorts should be conducted more comprehensively in the future under the supervision of child psychiatrists.

## Author contributions

**Conceptualization:** Mustafa Yaşar Özdamar, Şenol Biçer.

**Data curation:** Mustafa Yaşar Özdamar, Şenol Biçer.

**Formal analysis:** Mustafa Yaşar Özdamar, Şenol Biçer.

**Investigation:** Mustafa Yaşar Özdamar, Şenol Biçer.

**Methodology:** Mustafa Yaşar Özdamar, Şenol Biçer.

**Project administration:** Mustafa Yaşar Özdamar.

**Resources:** Mustafa Yaşar Özdamar, Şenol Biçer.

**Software:** Mustafa Yaşar Özdamar.

**Supervision:** Mustafa Yaşar Özdamar.

**Validation:** Mustafa Yaşar Özdamar.

**Visualization:** Mustafa Yaşar Özdamar.

**Writing—original draft:** Mustafa Yaşar Özdamar.

**Writing—review & editing:** Mustafa Yaşar Özdamar.
